# Molecular Insights into *Helicobacter pylori*-Induced Gastritis and Gastric Cancer

**DOI:** 10.3390/cancers18020331

**Published:** 2026-01-21

**Authors:** Silvia Salvatori, Irene Marafini, Pasquale De Vico, Antonio Fonsi, Giovanni Monteleone

**Affiliations:** 1 Department of Systems Medicine, University of Rome “Tor Vergata”, 00133 Rome, Italy; silvia.salvatori@ptvonline.it (S.S.); irene.marafini@ptvonline.it (I.M.); antonio.fonsi@ptvonline.it (A.F.); 2 Gastroenterology Unit, Fondazione Policlinico “Tor Vergata”, 00133 Rome, Italy; 3 Department of Anaesthesia, University of Rome “Tor Vergata”, 00133 Rome, Italy; pasquale.devico@ptvonline.it

**Keywords:** *H. pylori*, gastric cancer, chronic gastritis

## Abstract

*Helicobacter pylori* (*H. pylori*) is recognized as one of the most widespread and persistent bacterial infections globally, with a remarkable ability to colonize the human stomach. This pathogen is a major contributor to the development of gastric diseases, including gastric lymphoma and adenocarcinoma. The *H. pylori* infection triggers a complex pathogenic cascade within the gastric environment, characterized by prolonged inflammation and heightened oxidative stress, which fosters a milieu of immune dysregulation, where both innate and adaptive immune cells become activated inappropriately, thereby leading to epithelial injury and subsequent remodeling of the gastric tissue. As the infection persists, repeated cycles of inflammation and epithelial damage contribute to the development of epigenetic alterations, including changes in DNA methylation, histone modifications, and non-coding RNA expression, all of which render the gastric epithelium more susceptible to further aberrations, including dysplasia and cancer. In this article, we review the latest advances in understanding the molecular mechanisms of *H. pylori*-induced gastritis and its role in the progression of gastric cancer, offering new perspectives on the complex biology of this infection and its potential therapeutic implications for preventing the development of gastric malignancies.

## Simple Summary

*Helicobacter pylori* (*H. pylori*) is a widespread bacterium that infects the human stomach, contributing to various diseases, including peptic ulcers and gastric cancer. The infection triggers a long-lasting inflammatory response, which can eventually damage the stomach’s epithelial cells and promote genetic changes, such as alterations in DNA methylation and histone modifications that make the cells more likely to become cancerous. This review examines the molecular mechanisms behind *H. pylori*-induced gastritis and its role in the development of gastric cancer, highlighting how various immune cells and non-immune cells work together to create a disease-prone environment.

## 1. Introduction

*Helicobacter pylori* (*H. pylori*) is a spiral-shaped, Gram-negative bacterium that has evolved to colonize the acidic environment of the human stomach. Since its initial identification in the early 1980s, *H. pylori* has been recognized as the leading cause of chronic gastritis and remains the only bacterial species classified by the International Agency for Research on Cancer (IARC) as a Group I carcinogen [1]. Although many *H. pylori*-infected individuals never develop symptoms, a subset of them can experience serious complications, including peptic ulcers, gastric adenocarcinoma, and, less commonly, mucosa-associated lymphoid tissue (MALT) lymphoma [2,3,4,5,6]. Although pioneering epidemiological studies showed that *H. pylori* infected nearly half of the world’s population, recent data indicate a reduced trend in the global prevalence among adults. A recent comprehensive meta-analysis found that, between 2015 and 2022, the estimated global prevalence in adults was 43.9% (95% CI 42.3–45.5%), down from 52.6% before 1990 [7]. The same study found that in children and adolescents, the global prevalence remains high (around 35.1%) [7]. Furthermore, the Taipei Global Consensus II, based on studies published from 2016 to 2024, indicated that prevalence exceeded 60% among adults in Central Asia. Intermediate prevalence levels (30–60%) were observed in Eastern Europe, China, Russia, and the Americas, whereas the European regions showed the lowest prevalence (32.7%). Notably, the highest infection rates were reported among children in India, reaching 75% or higher [8]. These observations suggest that, although improvements in hygiene, socioeconomic conditions, and overall living standards have reduced adult *H. pylori* infection rates in many regions, the bacterium is probably acquired early in life and declines in pediatric or adolescent infection.

In line with the above data, the prevalence of peptic ulcer disease has declined in many parts of the world, particularly in developed countries [7]. Similarly, the global incidence of gastric cancer has generally decreased over recent decades, particularly in regions where *H. pylori* prevalence has fallen [9]. Nevertheless, the gastric cancer burden remains substantial. Among individuals born between 2008 and 2017, nearly 16 million gastric cancer cases are projected to occur over their lifetimes across 185 countries, and more than two-thirds of these are attributable to *H. pylori* infection [1,7,9]. Together, these epidemiological trends indicate that *H. pylori* remains the leading preventable cause of gastric cancer worldwide, and additional geographic, socioeconomic, and environmental factors, along with the intrinsic characteristics of infected subjects, can influence the *H. pylori* infection-associated carcinogenic risk [10].

In this narrative review, we summarize recent advances in understanding the molecular mechanisms underlying *H. pylori*-driven gastritis. In addition, we discuss emerging evidence explaining how *H. pylori*-induced gastritis contributes to the development of gastric cancer.

## 2. Molecular and Cellular Mechanisms of *H. pylori*-Induced Gastritis

*H. pylori* can persist within the gastric mucus layer despite the highly acidic environment, which inhibits the growth of most other bacteria. This survival mainly depends on the ability of *H. pylori* to synthesize elevated levels of urease, an enzyme that converts urea into carbon dioxide and ammonia, with the ammonia helping to neutralize the gastric acidity. *H. pylori* remains mainly within the mucus layer, although it can adhere to the gastric epithelial cells and, rarely, penetrate the mucosa [11].

**Figure 1:**
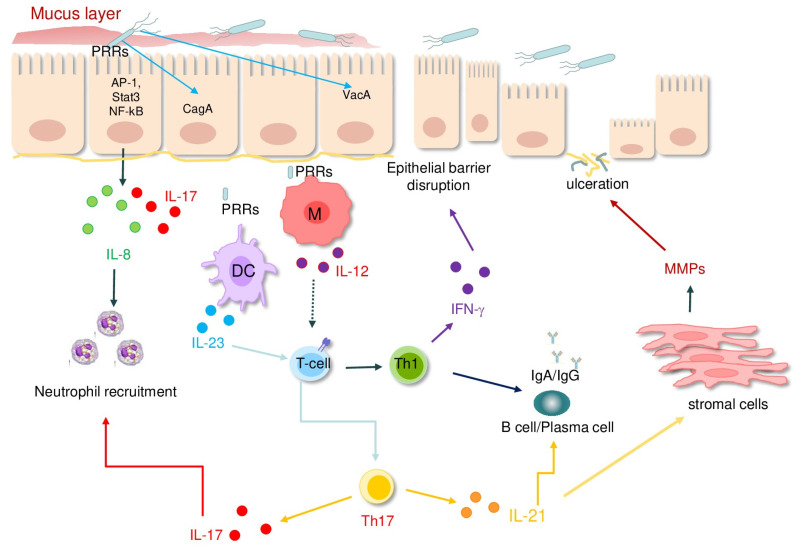
Schematic view of the main immune alterations documented in the *Helicobacter pylori*-infected gastric mucosa. Abbreviations: pattern recognition receptors (PPRs); macrophages (M); dendritic cells (DC); T helper cells (Th); interleukins (IL); interferon (IFN); matrix metalloproteinases (MMPs).

The pathogenesis of *H. pylori*-induced gastritis is multifactorial, involving a complex interplay of bacterial virulence factors and host immune responses that result in gastric mucosal inflammation and, eventually, epithelial cell injury and cancer, as illustrated in Figure 1.

Following adherence to the gastric epithelium, *H. pylori* is able to inject effector proteins into epithelial cells via its type IV secretion system (T4SS), thus altering their normal functions [12]. One of such proteins is the cytotoxin-associated gene A (CagA), which is then phosphorylated by Src kinases. This phenomenon leads to a deregulation of several intracellular signaling transduction pathways [e.g., Ras-Mitogen-Activated Protein Kinases (MAPK), signal transducer and activator of transcription (Stat)3, nuclear factor kappa-light-chain-enhancer of activated B cells (NF-kB)] [13,14,15,16], which promote changes to cell morphology, cell adhesion, and reorganization of the actin cytoskeleton, thus contributing to epithelial cell detachment and increased gastric permeability. Even in its unphosphorylated form, CagA can disrupt the E-cadherin/β-catenin complex, allowing β-catenin to translocate to the nucleus and activate oncogenic target genes, thereby promoting a gene expression profile favorable to tumor development [17]. In addition to CagA, *H. pylori* produces a variety of other virulence factors, including the Vacuolating cytotoxin A (VacA), which can facilitate the formation of anion-selective channels that disrupt mitochondrial membrane potential, impair ATP production, activate Bcl-2-associated X protein (BAX) and Bcl-2 antagonist/killer [BAK], promote cytochrome c release, and drive mitochondrial fragmentation thereby triggering apoptosis or mitochondrial dysfunction [18,19,20]. VacA also alters lysosomal and autophagy pathways to generate an intracellular niche that protects the bacteria from antibiotic treatment and leads to infection recrudescence after therapy [21,22]. Moreover, *H. pylori* can engage molecular dialog with host cells via extracellular vesicles, which can shuttle a broad range of bioactive molecules (e.g., proteins, nucleic acids, lipids, and metabolites) [23].

*H. pylori* infection triggers an initial innate immune response that activates pattern recognition receptors (PRRs) such as Toll-like receptors [TLRs] and nucleotide-binding oligomerization domain-like receptors (NLRs) on epithelial and immune cells [24]. This process triggers the secretion of pro-inflammatory cytokines, most notably interleukin-8 (IL-8) and IL-17, which attract neutrophils and macrophages to the site of infection [25,26,27,28]. These immune cells, in turn, release reactive oxygen species (ROS), proteases, and additional inflammatory cytokines that amplify the ongoing mucosal inflammation [29,30,31] (Figure 1). Moreover, pro-inflammatory cytokines can modulate stromal cell behavior, thus promoting the synthesis of extracellular matrix-degrading proteinases, which are involved in the formation of ulcers, and differentiation and function of cancer-associated fibroblasts (Figure 1) [32,33]. In the *H. pylori*-colonized mucosa, antigen-presenting cell-derived molecules, such as IL-12 and IL-23, stimulate the local differentiation of T helper (Th) type 1 and Th17 cells [25,33,34,35,36,37,38] (Figure 1), a phenomenon that is also sustained by defects in counter-regulatory mechanisms [39,40].

Host genetic factors are fundamental in shaping the response to *H. pylori*, and there is evidence that genetic predispositions not only contribute to the initial susceptibility to *H. pylori* infection but also influence the host’s ability to modulate the long-term consequences of *H. pylori*-induced gastritis. One of such genetic components is the variation in cytokine gene polymorphisms [41,42,43], which significantly affect the ability of the local immune system to respond effectively to *H. pylori* [44,45,46]. For instance, variations in the *IL-1β* gene can result in an exaggerated immune response, leading to more intense gastric inflammation and a higher likelihood of progressing to chronic gastritis or gastric cancer [47]. Similarly, changes in *IL-8* and *TNF-α* genes can alter the immune cell recruitment and the inflammatory response, further influencing disease outcomes [44,48,49], while SNPs in the *IL-10* gene (−819C/T, rs1800871) can determine a lower risk of GC [50]. HLA gene polymorphisms can also influence the outcome of the *H-pylori*-driven infection [51]. Furthermore, polymorphisms of the cytotoxin-associated gene A-related genes (e.g., *PTPN11* G/A at intron 3, rs2301756), those of genes involved in host immunity against *H. pylori* infection (e.g., *TLR4* +3725G/C, rs11536889), or polymorphisms of the genes essential for the differentiation of gastric epithelial cells (e.g., *RUNX3* T/A polymorphism at intron 3, rs760805) have been associated with gastric precancerous conditions [52].

*H. pylori* infection perturbs not only the gastric microbiome [53] but also the gut microbiome, thus contributing to a state of dysbiosis. For instance, *H. pylori* infection can reduce the diversity of gut microbiota, favoring the proliferation of harmful bacteria (e.g., Enterobacteriaceae, Clostridia, and Bacteroides) while suppressing beneficial species such as Lactobacillus and Bifidobacterium [54]. These alterations not only exacerbate gastric inflammation but may reduce the production of essential metabolites (e.g., short-chain fatty acids), alter gut permeability, and increase the susceptibility to infections and metabolic and autoimmune diseases (e.g., type 2 diabetes, cardiovascular diseases, neurological disorders, anemia, rheumatic disease, metabolic dysfunction-associated steatotic liver disease) [55,56,57,58,59,60,61]. A recent metagenomics (NGS) study confirmed the presence of dysbiosis in *H. pylori*-infected patients, characterized by a higher abundance of the *Bacteroidetes* and *Proteobacteria* phyla compared with controls. The same study also clarified functional alterations of the microbiome induced by *H. pylori*, including enrichment of pathways related to arginine and proline metabolism, cell cycle regulation, and MAPK signal transduction in the *H. pylori*-positive group [62]. Furthermore, microbial dysbiosis has been implicated in the initiation and progression of carcinogenesis by promoting a chronic inflammatory state, dysregulating both innate and adaptive immune responses, and inducing the release of microbial toxins and metabolites [63].

Consistently, eradication therapies targeting *H. pylori* can restore microbial diversity [64], even though there is evidence indicating that *H. pylori* eradication therapies could further alter the microbial composition [65]. Therefore, the long-term consequences of these interventions on gut microbiota and on the risk of carcinogenesis remain an area of active research. Similarly, further studies are needed to better ascertain the association between *H. pylori* and non-gastric digestive cancers (e.g., hepatocellular carcinoma, cholangiocarcinoma, and colorectal carcinoma) [66].

## 3. Molecular Mechanisms of *H. pylori*-Associated Gastric Cancer

**Figure 2:**
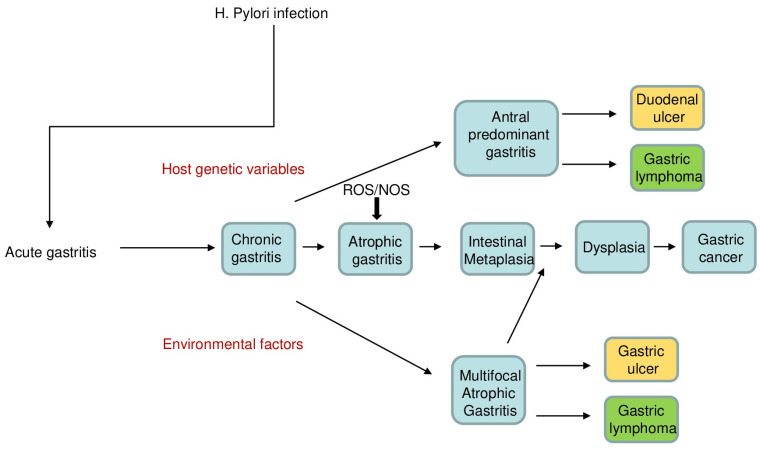
Schematic view of the main steps of gastric cancer. *Helicobacter* (*H.*) *pylori* infection causes acute gastritis that, in individuals with specific gene polymorphisms and in the presence of various dietary and socioeconomic variables, becomes chronic. In a subset of patients, chronic gastritis can favor the development of peptic (duodenal and gastric) ulcers or gastric lymphoma, depending on the predominant localization. Additional factors, such as reactive oxygen species (ROS)/nitrogen oxygen species (NOS), may promote the development of atrophic gastritis, thus triggering the pathogenic cascade leading to intestinal metaplasia, dysplasia, and carcinoma.

Gastric cancer is divided into two major histological types: intestinal and diffuse. Generally, the intestinal type is related to chronic *H. pylori* infection, whereas the diffuse type is mostly connected to genetic mutations and hereditary factors [5]. Additionally, the TCGA research team categorized gastric cancer into four molecular subtypes [i.e., Epstein–Barr virus (EBV)-positive, microsatellite unstable, genomically stable (GS), and chromosomal instability] to improve predictions of treatment response and patient outcomes [67]. A recent, retrospective, targeted sequencing of 1703 gastric tumor tissues revealed that 2.76% of samples were EBV-positive, 11.74% samples were *H. pylori*-positive, and 10 samples were positive for both. Most of the *H. pylori*-positive samples were genome stable (85.5%) and microsatellite stable (95%). Compared to GS tumors, mutations in *AKT3*, *EPAS1*, *MLH1*, and *BKT* and amplifications of *NFE2L2*, *TERC*, *MCL1*, and *TOP1* were significantly enriched in *H. pylori*-positive tumors. Moreover, compared to EBV-positive tumors, mutations in *PIK3CA*, *ARID1A*, and *PTEN* were significantly depleted in the *H. pylori*-positive subtype, while *TP53* mutations were enriched [68]. The processes by which *H. pylori* leads to gastric cancer are complex and involve multiple factors, as shown in Figure 2.

According to the Correa cascade, the invasive gastric carcinoma is preceded by a cascade of precancerous lesions [69]. As noted above, *H. pylori* infection leads to histologically active chronic inflammation, characterized by increased infiltration of the lamina propria with mononuclear leukocytes and neutrophils, as well as the formation of lymphoid aggregates and germinal centers. This condition may persist as non-atrophic chronic gastritis, with no glandular loss, or progress to multifocal atrophic gastritis (MAG), representing the initial step in the precancerous cascade [70,71]. MAG is marked by progressive changes in the gastric glands, which may eventually disappear. For example, metaplastic cells contain elevated levels of ornithine decarboxylase (ODC), an enzyme essential for cellular growth, which is regarded as a marker of premalignant transformation [72,73]. MAG may progress to intestinal metaplasia (IM), a phenotypic transformation in which gastric epithelial cells acquire an intestinal-like identity. IM initially appears as the ‘complete’ type (small intestine type), characterized by absorptive enterocytes with a brush border and digestive enzymes, interspersed with goblet and Paneth cells. It can later evolve into the ‘incomplete’ type (colonic type), marked by mucin-producing cells and the loss of both the brush border and intracellular digestive enzymes [74]. Incomplete IM is characterized by overexpression of genes involved in cell cycle regulation (e.g., COX-2 and cyclin D2) and is linked to an increased risk of gastric cancer [75]. IM can progress to dysplasia, initially low-grade and later high-grade (equivalent to carcinoma in situ). Histologically, dysplastic cells display enlarged, hyperchromatic, and crowded nuclei with frequent mitoses, all confined above the basement membrane, before ultimately advancing to invasive carcinoma [70].

For a detailed description of the signaling networks implicated in the *H. pylori*-driven gastric carcinogenesis, the reader is directed toward recent reviews [5,67].

Over time, the chronic inflammation and oxidative stress associated with *H. pylori* infection lead to genetic mutations and epigenetic alterations that drive the progression to gastric cancer. Notable genetic mutations observed in gastric cancer include alterations in the tumor suppressor gene p53, the oncogene c-MYC, and the cell cycle regulator cyclin D1, all of which disrupt normal cell cycle regulation and promote uncontrolled cell proliferation [76,77,78]. Moreover, *H. pylori* infection can cause chromosomal instability, resulting in aneuploidy and the loss of critical tumor suppressor genes such as CDH1, which encodes E-cadherin, a key molecule involved in cell–cell adhesion [79]. The loss of E-cadherin is a frequent event in gastric cancer and contributes to cancer cell invasion and metastasis by disrupting cellular adhesion [80].

One of the most deleterious consequences of chronic *H. pylori* infection is genotoxic stress. ROS and reactive nitrogen species (RNS), generated by persistent inflammation and through bacterial/host enzyme activity (e.g., via iNOS), cause a variety of DNA lesions, including strand breaks, base modifications (e.g., 8 oxo deoxyguanosine), and other oxidative adducts [81]. Experimental data have demonstrated that *H. pylori* infection induces DNA double-strand breaks (DSBs), often dependent on the T4SS machinery and involvement of host endonucleases (XPF/XPG) [79]. At the same time, *H. pylori* alters the host’s DNA repair capacity. For example, CagA-mediated disruption of signaling can impair DNA damage response factors (like p53, ATM/ATR) and suppress repair pathways such as base excision repair (BER) or mismatch repair (MMR) [82]. Additionally, *H. pylori* infection is associated with epigenetic modifications, including aberrant promoter methylation (e.g., of tumor suppressors), altered expression of microRNAs and long non-coding RNAs, all of which may silence protective genes (e.g., those involved in apoptosis or DNA repair) and shift the gene expression program toward carcinogenesis [83]. For instance, DNA hypermethylation of genes such as FOXD3 has been identified as an early epigenetic marker in *H. pylori*–related gastric carcinogenesis [84]. Additionally, METTL3, an enzyme involved in m6A methylation, influences the CXCL1/NF-κB signaling pathway in *H. pylori*-induced gastritis, thus increasing inflammatory responses and apoptosis in gastric cells [85]. *H. pylori* infection can also alter the expression of various miRNAs, including miR-21, which is frequently upregulated in gastric cancer. miR-21 promotes cell proliferation and survival while inhibiting apoptosis, making it a key player in cancer progression [86]. The *H. pylori*-colonized gastric epithelium over-expresses the proteinase-activated receptor (PAR) family members [87]. High expression of PAR2 is also seen in gastric cancer cells, where PAR-2 activation promotes the transactivation of the epidermal growth factor receptor (EGFR) signaling, a pathway involved in gastric cancer cell growth [88]. Moreover, studies in gastric adenocarcinoma cells showed that *H. pylori* by itself is sufficient to promote the expression and the activation of PAR-2 [89].

Another hallmark of *H. pylori*-induced gastric cancer is immune evasion. While the initial infection triggers an immune response, chronic inflammation suppresses the body’s ability to mount an effective anti-tumor immune response. Regulatory T cells, which are normally involved in maintaining immune tolerance and preventing autoimmunity, are recruited to the tumor microenvironment, where they suppress the activity of cytotoxic T cells. This immune suppression enables the tumor to escape immune surveillance, allowing cancer cells to persist and proliferate [90]. Furthermore, *H. pylori* has been shown to modulate immune checkpoint molecules such as programmed cell death protein 1 and cytotoxic T-lymphocyte-associated protein, which are normally involved in inhibiting T-cell activation. By upregulating these immune checkpoint proteins, *H. pylori* enhances immune evasion, providing tumor cells with an additional layer of protection from immune-mediated destruction [91]. Notably, the Epstein–Barr virus could induce immune evasion, thus amplifying the impact of the *H. pylori*-induced gastritis on the maintenance and differentiation of gastric cancer stem cells [92,93].

The chronic inflammation induced by *H. pylori* infection also creates a tumor-promoting microenvironment that supports angiogenesis (namely, the formation of new blood vessels), stromal remodeling, and the infiltration of immune cells, all of which contribute to tumor growth and metastasis [94]. In this inflammatory microenvironment, immune cells, such as macrophages and neutrophils, can secrete growth factors and cytokines that further drive tumor progression, making gastric cancer more aggressive and harder to treat [94].

During *H. pylori* infection, the activation of the NF-κB pathway enhances the expression of genes involved in cell proliferation, migration, and resistance to apoptosis [95]. These effects foster a pro-carcinogenic environment by promoting uncontrolled cell growth and survival. In addition, *H. pylori* infection triggers epithelial-to-mesenchymal transition (EMT), a process wherein epithelial cells lose their adhesion properties and acquire migratory and invasive characteristics. EMT is activated by signaling pathways such as TGF-β and Wnt/β-catenin, both of which are frequently dysregulated in gastric cancer [96]. Activated immune cells produce various cytokines that stimulate both immune and non-immune cells to produce matrix metalloproteinases, which promote the degradation of the extracellular matrix, thus creating a permissive environment for the invasion of cancer cells [33,97].

Together, these data indicate that *H. pylori* infection can promote several genetic and epigenetic alterations as well as activate a multitude of intracellular pathways that facilitate the gastric carcinogenic process.

## 4. *H. pylori*–Driven Metabolic Reprogramming

*H. pylori* infection induces profound and complex metabolic changes in the gastric mucosa, which play a crucial role in both the survival of the bacterium and the pathogenesis of associated gastric disorders. These alterations are driven by a combination of direct bacterial influence and the gastric inflammatory response [98]. One of the primary metabolic shifts induced by *H. pylori* infection is a reprogramming of cellular energy metabolism. In the gastric epithelial cells, there is a marked shift from oxidative phosphorylation towards increased glycolytic activity, even in the presence of oxygen, a phenomenon known as the Warburg effect, which is commonly associated with cancer cells [98]. This metabolic alteration relies, at least in part, on the production of urease that, as mentioned above, neutralizes gastric acid and creates a microenvironment permissive to *H. pylori*’s survival [99]. Moreover, *H. pylori* has been shown to modulate mitochondrial function by reducing oxidative phosphorylation and contributing to cellular energy deficits [100]. In experimental models, this mitochondrial dysfunction is associated with mitochondrial DNA damage and mutations, concomitant with the development of gastric intraepithelial neoplasia [101]. This reprogramming may impair the ability of the mucosa to effectively repair and regenerate, further promoting chronic inflammation.

*H. pylori* infection also exerts significant effects on lipid metabolism in the gastric mucosa. Infection leads to the overproduction of pro-inflammatory lipid mediators such as leukotrienes and prostaglandins, which are known to amplify the host’s immune response and contribute to tissue damage [102]. Additionally, *H. pylori* alters the composition of gastric mucus, changing the lipid profiles of mucus cells and potentially impairing the mucus layer’s ability to protect the underlying epithelial cells from acidic damage. This disruption may increase the susceptibility of the gastric mucosa to further damage and inflammation [103]. Moreover, recent studies suggest that *H. pylori* can hijack lipid metabolic pathways for its own benefit, facilitating membrane biosynthesis and sustaining its survival and virulence in the harsh gastric environment [104].

*H. pylori* infection influences amino acid metabolism, particularly the metabolism of glutamine, an amino acid that serves as a key energy source for both the bacterium and the host gastric epithelial cells. Glutamine is metabolized to produce glutamate and other intermediates, which can fuel both bacterial growth and mucosal cell survival [105]. The increased consumption of glutamine by infected cells and bacteria not only promotes microbial persistence but also facilitates the production of ROS and nitrogen species, leading to oxidative stress and inflammatory responses [106]. Furthermore, *H. pylori* modulates the urea cycle and ammonia metabolism in the gastric mucosa, leading to altered nitrogen balance and contributing to mucosal damage [107].

The metabolic shifts induced by *H. pylori* are tightly linked to the inflammatory and immune responses in the infected gastric mucosa. Inflammatory cytokines, such as IL-1β, TNF-α, and IL-6, promote further metabolic alterations. Additionally, immune cells such as neutrophils and macrophages, which are recruited to the site of infection, also undergo metabolic reprogramming, favoring glycolysis and a pro-inflammatory phenotype [98].

Recent research has also highlighted the involvement of specific molecular signaling pathways in the metabolic changes induced by *H. pylori*. The bacterium affects key metabolic regulators such as mTOR (mechanistic target of rapamycin), AMP-activated protein kinase (AMPK), and hypoxia-inducible factor 1α (HIF-1α), all of which play central roles in the regulation of cellular energy homeostasis [104]. By modulating these pathways, *H. pylori* effectively alters host cell metabolism to support its survival and virulence, while simultaneously exacerbating the inflammatory milieu. The interplay between *H. pylori*-induced metabolic reprogramming and host immune responses may create a vicious cycle that drives disease progression and contributes to the development of more severe conditions such as gastric cancer.

## 5. Implications of the Correa Cascade

The Correa cascade, a well-established framework that describes the multistep process by which *H. pylori* infection leads to the development of gastric cancer [70], highlights the importance of early detection and prevention in reducing the incidence of gastric cancer (Figure 2).

Recent evidence emphasizes that early detection and eradication of *H. pylori* can significantly reduce the risk of developing gastric cancer, especially in high-risk populations [8]. The clinical management of *H. pylori* infection has undergone significant transformation over recent decades, primarily due to the introduction and widespread use of antibiotic-based eradication therapies. These treatments have proven remarkably effective not only in clearing the infection but also in preventing the development of related complications [108]. However, despite these positive outcomes, the rising issue of antibiotic resistance has become a formidable challenge in the treatment of *H. pylori* infection [109,110]. Over time, the bacterium has adapted to the selective pressures exerted by commonly used antibiotics, rendering some of the standard treatments less effective. This growing resistance underscores the urgent need for continuous research to identify novel therapeutic approaches and to refine existing treatment protocols [111].

Not all individuals with *H. pylori* infection will progress to cancer, and genetic factors (e.g., variations in the immune response genes) and environmental influences (e.g., diet, smoking, and socioeconomic status) can modulate the risk of progression [112]. This variability suggests that a personalized approach to managing *H. pylori* infection, including monitoring for early signs of precancerous lesions and regular screening, may be beneficial in high-risk populations.

## 6. Conclusions

The persistence of *H. pylori* as a significant global health concern emphasizes the complexity of its pathogenic mechanisms and the multifactorial nature of its long-term health outcomes. Over the past few decades, our understanding of the molecular and cellular mechanisms of *H. pylori* infection has expanded significantly, shedding light on its intricate role in the pathogenesis of gastric diseases and, particularly, gastric cancer.

New technological approaches, such as single-cell RNA sequencing (scRNA-seq) and multi-omics strategies, are emerging as powerful tools to dissect gene expression signatures underlying *H. pylori* infection. Using scRNA-seq technology, Hu and colleagues have recently documented the ability of *H. pylori* to promote an immunosuppressive microenvironment that facilitates the bacterium’s persistence [113]. Furthermore, a multi-omics study investigating the molecular mechanisms connecting *H. pylori* infection to the different stages of gastric pathology identified several genes that may help predict *H. pylori*-associated gastric cancer risk and patient survival [114].

From an epidemiological standpoint, while the global prevalence of *H. pylori* has declined, particularly in developed countries, the burden of gastric cancer remains high, especially in low- and middle-income countries [1]. The long latency period between infection and cancer development poses challenges for early detection, particularly in populations that may not have access to regular screening. Additionally, the rise in antibiotic resistance could hamper treatment strategies, suggesting the need for novel antibiotics or alternative therapies [109,110]. In this context, encouraging results may come from the use of specific bioactive compounds from aloe vera that inhibit *H. pylori* neutrophil-activating protein, as well as fisetin, which targets the *H. pylori* HtrA protease [115,116].

The growing recognition of the bacterial strain-specific differences in virulence and the contribution of host genetics to disease outcomes further suggests that a one-size-fits-all approach may not be sufficient for managing *H. pylori* infection and preventing gastric cancer. Instead, a more personalized approach that takes into account genetic risk factors, bacterial strain type, and local epidemiological trends will be crucial in refining treatment strategies [117]. Finally, in the precancerous lesions of gastric cancer, *H. pylori* eradication may fail to effectively suppress chronic inflammation or halt the progression of Correa’s cascade [118], suggesting the need for new promising approaches targeting specific pathways to treat chronic atrophic gastritis and intestinal metaplasia [119].

Therefore, further research is needed to clarify strain-specific pathways and host interactions, to predict the dynamic progression of inflammation toward tumorigenesis, to optimize early eradication strategies to prevent persistent epigenetic alterations, and ultimately to reduce long-term cancer risk.

## Data Availability

No new data were created or analyzed in this study. Data sharing is not applicable to this article.
